# Late presentation of vesicoureteral reflux: An unusual cause of pyelonephritis in adults

**DOI:** 10.1016/j.ijscr.2018.10.058

**Published:** 2018-10-29

**Authors:** Bernardo Pereira, Catarina Macedo, Sara Anacleto, Marina Gonçalves, Estêvão Lima, Emanuel Carvalho-Dias

**Affiliations:** a USF Ruães, ACeS Cávado I – Braga, Braga, Portugal; b USF Pró-Saúde, ACeS Cávado II, Gerês/Cabreira, Braga, Portugal; c Urology Department, Hospital de Braga, Braga, Portugal; d Life and Health Sciences Research Institute (ICVS), School of Medicine, University of Minho, Braga, Portugal

**Keywords:** VUR, vesicoureteral reflux, VCUG, voiding cysourethrogram, UTI, urinary tract infection, CT, computerized tomography, US, ultrasound, Adult vesicoureteral reflux, Recurrent pyelonephritis, Bulking agent

## Abstract

Introduction: Vesicoureteral reflux (VUR) corresponds to the reflux of urine from the bladder into the upper urinary system. It can be a congenital or an acquired anomaly and although its incidence is high in children it is uncommon in the adult life. One of its presentations in the adult population is the presence of recurrent Pyelonephritis.

Case presentation: Here we report a case of an adult patient with repetitive uncomplicated pyelonephritis caused by VUR. VUR was successfully managed endoscopically with subureteral injection of a bulking agent. A literature review of adult presenting VUR was performed.

Discussion: The first presentation of VUR in the adult life is rare. One of the most typical presentation is the presence of recurrent uncomplicated Pyelonephritis. Although no guidelines exists to study the presence of VUR in adult patients with Pyelonephritis, in the presence of several recurrent episodes of Pyelonephritis we should think in VUR as a possible cause. Even in adults, endoscopic management of VUR is an effective treatment with low morbidity.

Conclusion: VUR can first present in the adult life, with recurrent episodes of UTI. The diagnosis is a suspicious one and is confirmed by VUCG. VUR in adults can be effectively managed with endoscopic injection of bulking agents.

## Highlights

• Vesicoureteral reflux presenting for the first time in adults is rare.
• The diagnosis should be suspicious in adult patients with recurrent Pyelonephritis without recognizable cause.
• Endoscopic treatment with bulking agents is a minimally invasive technique with good results for adult vesicoureteral reflux.

## 1 Introduction

Vesicoureteral reflux (VUR) corresponds to the reflux of urine from the bladder into the upper urinary system and can be classified as congenital (due to ureterovesical junction anomaly) or secondary (due to lower urinary tract dysfunction). VUR is one the most common urologic diseases in children, with an incidence of 0,5 to 3% in normal children and 30–40% in children with urinary tract infection (UTI) [1]. The clinical significance of VUR is mainly due to the fact that VUR can cause recurrent Pyelonephritis with consequent irreversible renal damage, hypertension and renal failure. The incidence of VUR associated with UTI decrease significantly in children after 5 years-old and its first presentation in adults is rare but the exact incidence is unknown.

Pyelonephritis is a very common disease in adults with an estimated 10.5 million to 25.9 million cases worldwide annually and imaging studies, mainly ultrasound or CT scan, are reserved for complicated cases (worsening or lack of improvement after 24–48 hours of therapy) to assess for obstruction, abscess, or necrotizing infection [2]. However, the gold standard for diagnosis of VUR is voiding cystourethrogram (VCUG) [3], and for adult patients with pyelonephritis no guidelines exist with indications to perform VCUG. Here, we report a case of recurrent UTI in a young female caused by VUR first presenting in the adult life. This case has been reported in line with the SCARE criteria [4].

## 2 Case report

**Fig. 1:**
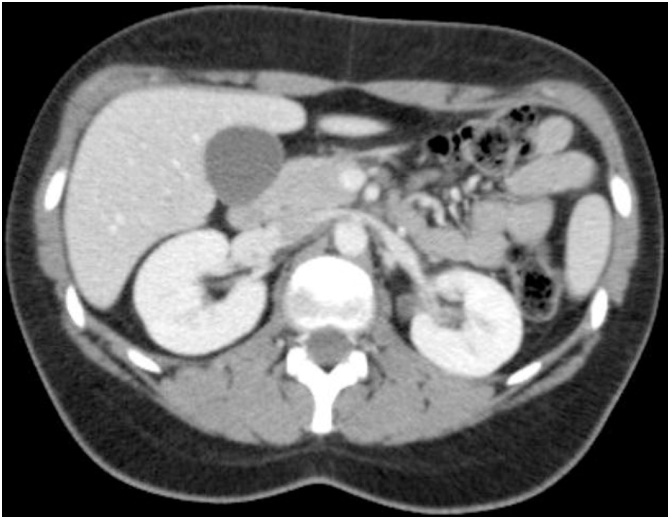
CT scan, axial view, showing absence of hydronephrosis bilateral.

**Fig. 2:**
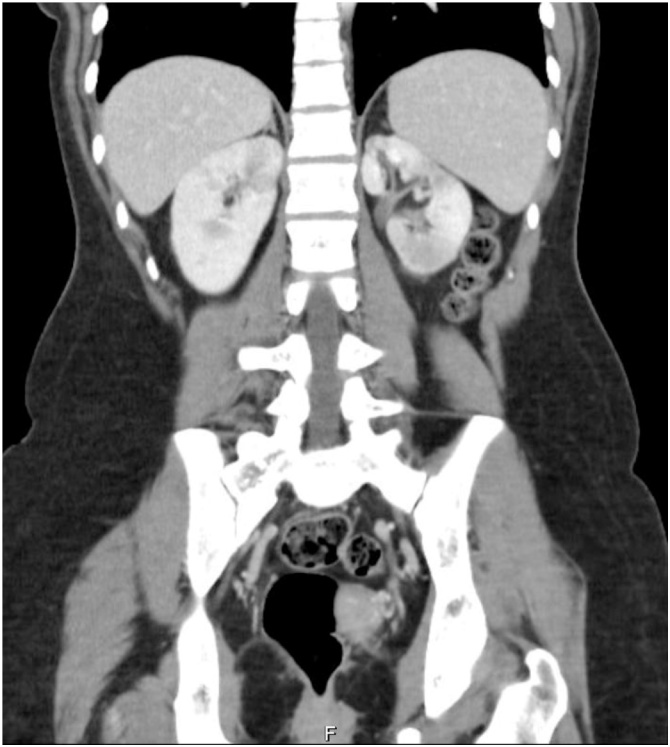
CT scan, sagital view, showing a heterogeneous uptake of intravenous contrast in left kidney in favor of pyelonephritis.

A 20 year-old woman with no relevant past medical or surgical history, recurred to the emergency department of our institute because of left flank pain and fever. On physical examination tenderness at percussion of left lumbar region was observed, the pulse was 90 beats per minute, and the blood pressure 115/75 mmHg. The with-cell count was 14,200 per cubic millimeter, the plasmatic creatinine concentration was 0,9 mg per deciliter and urinalysis was positive for nitrites. The patient was discharged with the diagnosis of uncomplicated left Pyelonephritis and treated with a 7-day regimen of levofloxacine. The patient was completely asymptomatic after completing the treatment, however in the next 12 months she developed 10 episodes of recurrent non-complicated left-sided Pyelonephritis. In all the episodes a urine culture revealed more than 10,000 colony-forming units of *Escherichia coli* per milliliter of urine. The US examination of kidney and bladder revealed no alterations and the contrast enhanced CT scan performed at emergency in one of the episodes revealed a heterogeneous uptake of intravenous contrast in left kidney in favor of pyelonephritis, but absence of urinary system obstruction (Fig. 1, Fig. 2).

**Fig. 3:**
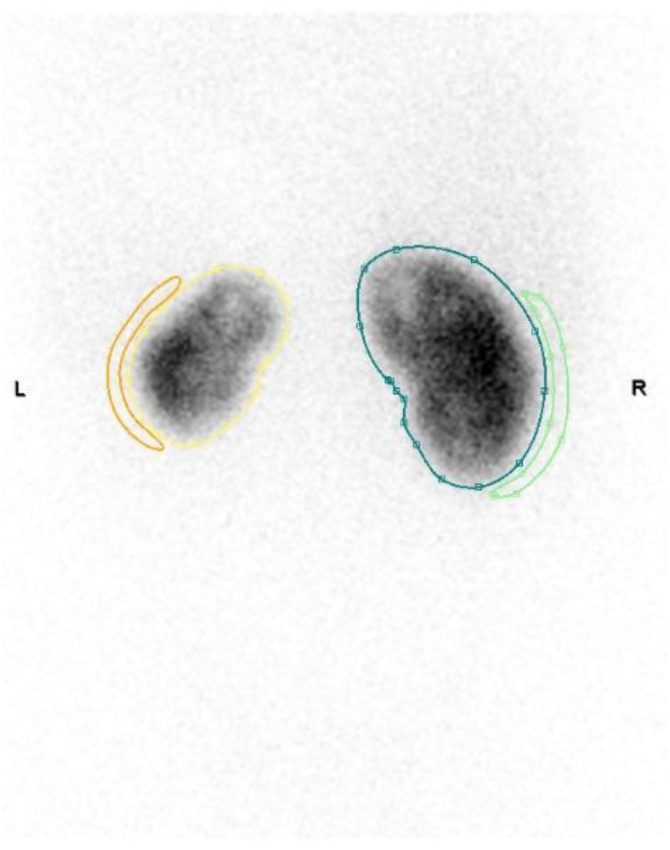
Renal DMSA scan showing a left kidney with decreased uptake of DMSA with several cortical lesions.

**Fig. 4:**
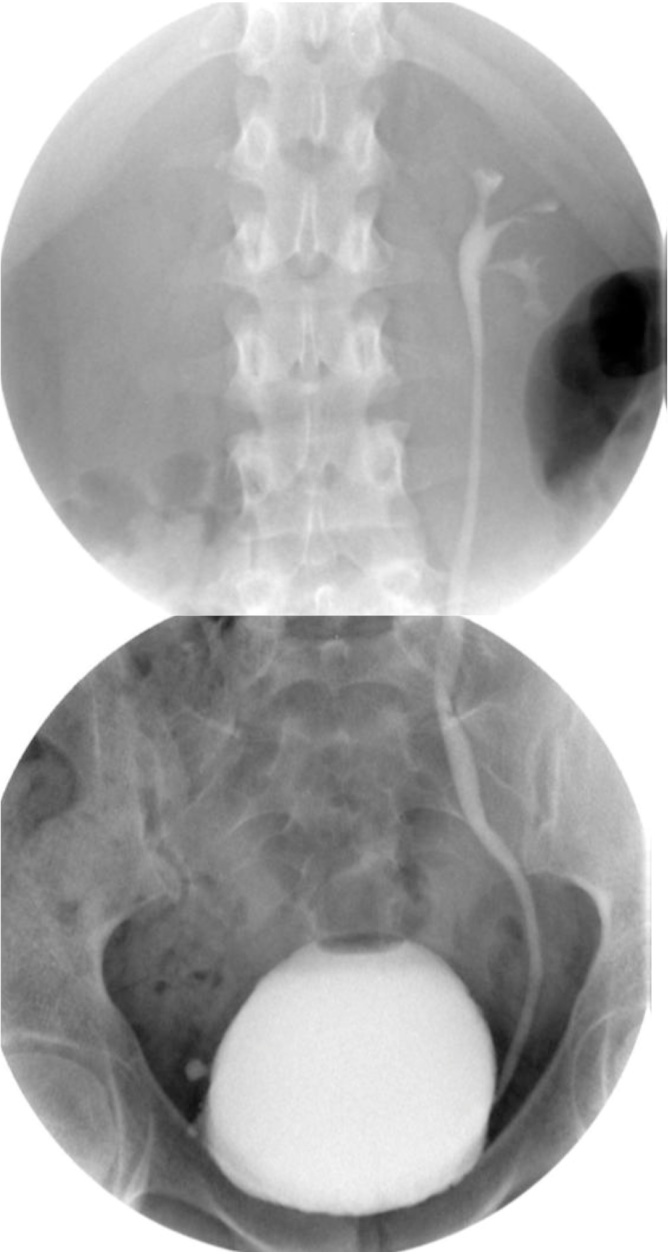
VCUG revealed a left sided grade II VUR.

The patient was referred for our Urologic Department for evaluation of recurrent Pyelonephritis and a prophylactic antibiotic regimen of nitrofurantoin 100 mg once a day was prescribed. The urologic evaluation was completed with a renal DMSA scan and VCUG. Renal DMSA scan revealed a left kidney with decreased uptake of DMSA with several cortical lesions. The differential kidney function was 70% for right kidney and 30% for the left kidney (Fig. 3) and the VCUG revealed a left grade II VUR (Fig. 4). The patient was submitted to endoscopic treatment of left sided VUR with subureteric injection of dextranomer/hyaluronic acid copolymer (Deflux®). The procedure was uneventful and post-operative VCUG revealed complete resolution of VUR. After 6 months of endoscopic treatment the patient is completely asymptomatic without any report of Pyelonephritis.

## 3 Discussion

Vesicoureteral reflux (VUR) may be congenital or acquired. The most frequent is primary VUR, which is mostly diagnosed by the presence of hydronephrosis during prenatal screening ultrasound or during the investigation of urinary tract infection in children [3]. When present, VUR is the most significant risk factor in the etiology of pyelonephritis [5]. The first presentation of VUR in adults is rare. So, there are very few studies on the relationship between acute pyelonephritis and VUR in the adult population, compared with studies on children. Choi et al. showed that VUR was observed in only 2,3% of adult women with uncomplicated acute pyelonephritis [6]. According to that, VUR does not seem to be an important cause of uncomplicated acute pyelonephritis in adults. One possible explication is that reflux persisting into adulthood is usually low grade, with high-grade VUR being generally diagnosed and treated earlier in life [2]. Yet, the prevalence of VUR in adulthood seems to be higher in women [7]. A critical point is that no guidelines exists indicating the circumstances in which VUR should be investigated in adults with uncomplicated Pyelonephritis.

The first presentation and clinical evolution of VUR in adults is significantly different from that presenting during childhood. Adult presenting VUR is usually symptomatic with recurrent episodes of UTI or flank pain. In children, the spontaneous resolution of VUR is dependent on age of presentation, sex, grade, laterality, clinical presentation, and anatomy. The presence of renal cortical abnormality, bladder dysfunction, high grade of reflux and breakthrough febrile UTIs are negative predictive factors for reflux resolution. However, the spontaneous resolution observed in children is not expected in adults. In adults to prevent symptomatic VUR, particularly UTIs, reflux nephropathy and hypertension, the treatment is usually mandatory [8]. However, there is no conclusive evidence regarding VUR correction in adults.

Although ureteral reimplantation remains the standard surgical treatment of VUR, the success rate in the adult population is lower than in children [7]. Additionally, the demand for a reduction of the surgical morbidity has driven the search for an alternative technique. Endoscopic treatment with bulking agents for VUR, has become an accepted alternative to ureteral reimplantation because of limited and minor complication rates, fast recovery, and low costs related to the procedure. Some reports have documented that endoscopic injection of dextranomer/hyaluronic acid copolymer (Deflux^®^) or polydimethylsiloxane for VUR is an effective treatment after puberty with minimal morbidity. Moore et al. showed an overall ureteral cure rate after a single injection of 93% with resolution of VUR in 89% of the 27 postpubertal patients treated [7], but the overall success rate after a single endoscopic injection in adult series has varied greatly from 51% to 91%, depending on the bulking agent used and the population characteristics [[8], [9], [10], [11], [12], [13]].

## 4 Conclusion

VUR is a very common urologic disease during childhood, but the first clinical presentation in adulthood is rare. However, in case of recurrent urinary tract infections (UTIs) and pyelonephritis in adult population it is important to keep this disease in the differential diagnosis, because if undiagnosed VUR may cause severe consequences for patients mainly arterial hypertension and renal failure. Even in adults, endoscopic treatment is effective for complete resolution of symptomatic VUR with minimal morbidity.

## Conflict of interest

Authors have no conflict of interest to declare.

## Funding

None.

## Ethical approval

Case report no ethical approval needed at our institution

## Author contribution

All authors contributed in data collection, data analysis and interpretation, and writing the paper.

## Consent

Written informed consent was obtained from patient for publication of this case report. A copy of written consent is available for review by the Editor-in-chief of this journal on request. 5

Patient signed an informed consent

## Provenance and peer review

Not commissioned, externally peer reviewed.

## Registration of research studies

Case report.

## Guarantor

Emanuel Carvalho-Dias.
